# Supplementary material for: Dynamic behavior of the locus coeruleus during arousal-related memory processing in a multi-modal 7T fMRI paradigm
Source: eLife. 2020 Jun 24;9:e52059. doi: 10.7554/eLife.52059 (PMC7343392; doi:10.7554/eLife.52059)
Supplement: Supplementary file 1. [file elife-52059-supp1.docx]

**Supplementary File 1:** Demographics and neuropsychological performance of the group (N=27)

|  | Median | IQR | Possible range |
| --- | --- | --- | --- |
| Age (years) | 22 | 19.5 - 23.5 | 20 - 30 |
| Education (years) | 16 | 16 - 16 | 13 - 20 |
| Hamilton Depression Rating Scale | 1 | 0 - 2.5 | 0 - 30 |
| 15-Word Learning Test | 58 | 49 - 63.5 | 0 - 75 |
| Delayed Word Learning | 13 | 9.5 - 14.5 | 0 - 15 |
| Word Learning Recognition | 30 | 29 - 30 | 0 - 30 |
| Digit span Forward | 10 | 8 - 11 | 0 - 14 |
| Digit span Backwards | 8 | 6 - 10 | 0 - 12 |
| Verbal Fluency Test Animal | 26 | 22 - 29.5 | 0 - 36 |
| Verbal Fluency - Profession | 20 | 17.5 - 21.5 | 0 - 36 |
| Verbal Fluency - letter M | 16 | 12 - 19.5 | 0 -36 |
| Letter Digit Symbol Substitution Test | 63 | 61 - 69 | 0 - 125 |
| Perceived Stress Scale | 8 | 6 - 12 | 0 - 40 |
