# Supplementary material for: Dynamic behavior of the locus coeruleus during arousal-related memory processing in a multi-modal 7T fMRI paradigm
Source: eLife. 2020 Jun 24;9:e52059. doi: 10.7554/eLife.52059 (PMC7343392; doi:10.7554/eLife.52059)
Supplement: Supplementary file 2. — Note: numbers provided are N and % compared to the total sample size (N = 27); HRV = heart rate variability [file elife-52059-supp2.docx]

**Supplementary File 2:** Available data per modality and time point after pre-processing.

|  | Baseline (N (%)) | Encoding (N (%)) | Consolidation (N (%)) | Recollection (N (%)) |
| --- | --- | --- | --- | --- |
| BOLD fMRI | 24 (88.89 %) | 24 (88.89 %) | 24 (88.89 %) | 24 (88.89 %) |
| HRV | 22 (81.48 %) | 22 (81.48 %) | 21 (77.78 %) | 23 (85.19 %) |
| sAA change | 17 (62.96 %) | 16 (59.26 %) | 18 (66.69 %) | 17 (62.96 %) |
| All modalities | 13 (48.15 %) | 12 (44.44 %) | 14 (51.85 %) | 15 (55.56 %) |

Note: numbers provided are N and % compared to the total sample size (N=27); HRV= heart rate variability
