# Supplementary material for: Dynamic behavior of the locus coeruleus during arousal-related memory processing in a multi-modal 7T fMRI paradigm
Source: eLife. 2020 Jun 24;9:e52059. doi: 10.7554/eLife.52059 (PMC7343392; doi:10.7554/eLife.52059)
Supplement: Supplementary file 3. — Note: Linear mixed effects models with random intercept for each person and task stage as fixed effect. Estimates indicate the unstandardized beta-coefficients. P-values are adjusted for multiple comparisons using the False Discovery rate. [file elife-52059-supp3.docx]

**Supplementary File 3**: Task stages differences in sAA, rMSSD or LC BOLD variance

| **Task contrast** | **Estimate** | **t-value** | **p-value** | **95% CI** |
| --- | --- | --- | --- | --- |
| 1. **ΔsAA** | | | | |
| Encoding - Baseline | -27.826 | -1.210 | 0.279 | [-91.374, 35.723] |
| Consolidation - Baseline | 38.119 | 1.626 | 0.214 | [-26.483, 102.721] |
| Recollection - Baseline | 9.210 | 0.360 | 0.720 | [-61.03, 79.45] |
| Encoding - Consolidation | -65.945 | -2.907 | **0.033** | [-128.369, -3.521] |
| Encoding - Recollection | -37.035 | -1.490 | 0.214 | [-105.288, 31.218] |
| Consolidation - Recollection | -37.035 | -1.490 | 0.214 | [-105.288, 31.218] |
| 1. **rMSSD** | | | | |
| Encoding - Baseline | 22.782 | 1.769 | 0.162 | [-12.182, 57.745] |
| Consolidation - Baseline | 5.747 | 0.439 | 0.794 | [-29.754, 41.247] |
| Recollection - Baseline | -0.217 | -0.017 | 0.987 | [-35.37, 34.937] |
| Encoding - Consolidation | 17.035 | 1.303 | 0.295 | [-18.466, 52.535] |
| Encoding - Recollection | 22.998 | 1.775 | 0.162 | [-12.155, 58.152] |
| Consolidation - Recollection | 22.998 | 1.775 | 0.162 | [-12.155, 58.152] |
| 1. **LC variance** | | | | |
| Encoding - Baseline | -28.696 | -0.048 | 0.962 | [-1656.557, 1599.164] |
| Consolidation - Baseline | 1218.127 | 2.030 | 0.138 | [-409.733, 2845.988] |
| Recollection - Baseline | 819.622 | 1.366 | 0.211 | [-808.238, 2447.482] |
| Encoding - Consolidation | -1246.824 | -2.078 | 0.138 | [-2874.684, 381.037] |
| Encoding - Recollection | -848.318 | -1.414 | 0.211 | [-2476.179, 779.542] |
| Consolidation - Recollection | -848.318 | -1.414 | 0.211 | [-2476.179, 779.542] |

Note: Linear mixed effects models with random intercept for each person and task stage as fixed effect. Estimates indicate the unstandardized beta-coefficients. P-values are adjusted for multiple comparisons using the False Discovery rate.
