# Supplementary material for: Dynamic behavior of the locus coeruleus during arousal-related memory processing in a multi-modal 7T fMRI paradigm
Source: eLife. 2020 Jun 24;9:e52059. doi: 10.7554/eLife.52059 (PMC7343392; doi:10.7554/eLife.52059)
Supplement: Supplementary file 4. — Note: Linear mixed effects models with random intercept for each person, task stage, frequency and their interaction as fixed effect. Estimates indicate the unstandardized beta-coefficients. P-values are adjusted for multiple comparisons using the False Discovery rate. [file elife-52059-supp4.docx]

**Supplementary File 4**: Relationship between frequency and coherence between LC and heart rate variability across the task stages (Spatially normalized pipeline)

| **Task contrast** | **Estimate** | **t-value** | **p-value** | **95% CI** |
| --- | --- | --- | --- | --- |
| **LC** | | | | |
| Frequency: Baseline - Consolidation | 0.013 | 0.413 | 0.976 | [-0.067, 0.093] |
| Frequency: Baseline - Encoding | 0.413 | 1.789 | 0.418 | [-0.024, 0.135] |
| Frequency: Baseline - Recollection | 0.976 | -1.826 | 0.418 | [-0.134, 0.023] |
| Frequency: Consolidation - Encoding | 0.055 | 1.355 | 0.633 | [-0.038, 0.123] |
| Frequency: Consolidation - Recollection | 1.789 | -2.222 | 0.352 | [-0.148, 0.011] |
| Frequency: Encoding - Recollection | 0.418 | -3.636 | **0.010** | [-0.19, -0.033] |
| **Reference** | | | | |
| Frequency: Baseline - Consolidation | -0.029 | -0.871 | 0.984 | [-0.114, 0.056] |
| Frequency: Baseline - Encoding | -0.124 | -3.773 | **0.006** | [-0.208, -0.039] |
| Frequency: Baseline - Recollection | -0.116 | -3.523 | **0.007** | [-0.2, -0.031] |
| Frequency: Consolidation - Encoding | -0.095 | -2.858 | **0.044** | [-0.18, -0.01] |
| Frequency: Consolidation - Recollection | -0.087 | -2.611 | **0.067** | [-0.172, -0.001] |
| Frequency: Encoding - Recollection | 0.008 | 0.250 | 0.994 | [-0.076, 0.092] |

Note: Linear mixed effects models with random intercept for each person, task stage, frequency and their interaction as fixed effect. Estimates indicate the unstandardized beta-coefficients. P-values are adjusted for multiple comparisons using the False Discovery rate.
