# Supplementary material for: Dynamic behavior of the locus coeruleus during arousal-related memory processing in a multi-modal 7T fMRI paradigm
Source: eLife. 2020 Jun 24;9:e52059. doi: 10.7554/eLife.52059 (PMC7343392; doi:10.7554/eLife.52059)
Supplement: Supplementary file 5. — Note: Linear mixed effects models with random intercept for each person, task stage, frequency and their interaction as fixed effect. Estimates indicate the unstandardized beta-coefficients. P-values are adjusted for multiple comparisons using the False Discovery rate. [file elife-52059-supp5.docx]

**Supplementary File 5**: Relationship between frequency and coherence between LC and heart rate variability across the task stages (FIXed pipeline)

| **Task contrast** | **Estimate** | **t-value** | **p-value** | **95% CI** |
| --- | --- | --- | --- | --- |
| **LC** | | | | |
| Frequency: Baseline - Consolidation | -0.121 | -4.112 | **< 0.001** | [-0.197, -0.045] |
| Frequency: Baseline - Encoding | -0.043 | -1.466 | 0.487 | [-0.118, - 0.032] |
| Frequency: Baseline - Recollection | -0.163 | -5.643 | **< 0.001** | [-0.237, -0.089] |
| Frequency: Consolidation - Encoding | 0.079 | 2.663 | **0.058** | [0.003, 0.154] |
| Frequency: Consolidation - Recollection | -0.041 | -1.419 | 0.487 | [-0.116, 0.034] |
| Frequency: Encoding - Recollection | -0.120 | -4.161 | **< 0.001** | [-0.194, -0.046] |
| **Reference** | | | | |
| Frequency: Baseline - Consolidation | -0.031 | -0.990 | 0.859 | [-0.11, 0.049] |
| Frequency: Baseline - Encoding | -0.055 | -1.800 | 0.410 | [-0.133, 0.023] |
| Frequency: Baseline - Recollection | -0.117 | -3.892 | **0.003** | [-0.195, -0.04] |
| Frequency: Consolidation - Encoding | -0.024 | -0.789 | 0.859 | [-0.104. 0.055] |
| Frequency: Consolidation - Recollection | -0.087 | -2.845 | 0.069 | [-0.165, -0.008] |
| Frequency: Encoding - Recollection | -0.063 | -2.072 | 0.325 | [-0.14, 0.015] |

Note: Linear mixed effects models with random intercept for each person, task stage, frequency and their interaction as fixed effect. Estimates indicate the unstandardized beta-coefficients. P-values are adjusted for multiple comparisons using the False Discovery rate.
