# Supplementary material for: Dynamic behavior of the locus coeruleus during arousal-related memory processing in a multi-modal 7T fMRI paradigm
Source: eLife. 2020 Jun 24;9:e52059. doi: 10.7554/eLife.52059 (PMC7343392; doi:10.7554/eLife.52059)
Supplement: Supplementary file 6. — Note: Linear mixed effects models with random intercept for each person, task stage, frequency and their interaction as fixed effect. Estimates indicate the unstandardized beta-coefficients. P-values are adjusted for multiple comparisons using the False Discovery rate. [file elife-52059-supp6.docx]

**Supplementary File 6** Relationship between frequency and coherence between LC and heart rate variability across the task stages (Fixed + Explicit Resp pipeline)

| **Task contrast** | **Estimate** | **t-value** | **p-value** | **95% CI** |
| --- | --- | --- | --- | --- |
| **LC** | | | | |
| Frequency: Baseline - Consolidation | -0.155 | -5.267 | **< 0.001** | [-0.23, -0.079] |
| Frequency: Baseline - Encoding | -0.059 | -2.022 | 0.216 | [-0.133, 0.016] |
| Frequency: Baseline - Recollection | -0.196 | -6.807 | **< 0.001** | [-0.27, -0.122] |
| Frequency: Consolidation - Encoding | 0.096 | 3.303 | **0.008** | [0.021, 0.171] |
| Frequency: Consolidation - Recollection | -0.041 | -1.427 | 0.482 | [-0.115, 0.033] |
| Frequency: Encoding - Recollection | -0.137 | -4.819 | **< 0.001** | [-0.21, -0.064] |
| **Reference** | | | | |
| Frequency: Baseline - Consolidation | 0.010 | 0.327 | 0.988 | [-0.068, 0.088] |
| Frequency: Baseline - Encoding | -0.056 | -1.876 | 0.358 | [-0.133, 0.021] |
| Frequency: Baseline - Recollection | -0.102 | -3.428 | **0.010** | [-0.178, -0.025] |
| Frequency: Consolidation - Encoding | -0.066 | -2.182 | 0.257 | [-0.144, 0.012] |
| Frequency: Consolidation - Recollection | -0.111 | -3.719 | **0.007** | [-0.189, -0.034] |
| Frequency: Encoding - Recollection | -0.045 | -1.532 | 0.502 | [-0.122, 0.031] |

Note: Linear mixed effects models with random intercept for each person, task stage, frequency and their interaction as fixed effect. Estimates indicate the unstandardized beta-coefficients. P-values are adjusted for multiple comparisons using the False Discovery rate.
