# Supplementary material for: Dynamic behavior of the locus coeruleus during arousal-related memory processing in a multi-modal 7T fMRI paradigm
Source: eLife. 2020 Jun 24;9:e52059. doi: 10.7554/eLife.52059 (PMC7343392; doi:10.7554/eLife.52059)
Supplement: Supplementary file 7. — Note: Linear mixed effects models with random intercept for each person, task stage, frequency and their interaction as fixed effect. Estimates indicate the unstandardized beta-coefficients. P-values are adjusted for multiple comparisons using the False Discovery rate. [file elife-52059-supp7.docx]

**Supplementary File 7**: Relationship between frequency and coherence between LC and heart rate variability across the task stages (Fixed + Explicit Phys pipeline)

| **Task contrast** | **Estimate** | **t-value** | **p-value** | **95% CI** |
| --- | --- | --- | --- | --- |
| 1. **LC** | | | | |
| Frequency: Baseline - Consolidation | -0.197 | -6.647 | **< 0.001** | [-0.273, -0.121] |
| Frequency: Baseline - Encoding | -0.069 | -2.361 | 0.102 | [-0.144, 0.006] |
| Frequency: Baseline - Recollection | -0.190 | -6.552 | **< 0.001** | [-0.264, -0.115] |
| Frequency: Consolidation - Encoding | 0.128 | 4.314 | **< 0.001** | [0.052, 0.204] |
| Frequency: Consolidation - Recollection | 0.007 | 0.245 | 0.995 | [-0.068, 0.083] |
| Frequency: Encoding - Recollection | -0.121 | -4.165 | **< 0.001** | [-0.195, -0.046] |
| 1. **Reference** | | | | |
| Frequency: Baseline - Consolidation | -0.035 | -1.138 | 0.793 | [-0.115, 0.044] |
| Frequency: Baseline - Encoding | -0.064 | -2.085 | 0.259 | [-0.143, 0.015] |
| Frequency: Baseline - Recollection | -0.126 | -4.150 | **0.001** | [-0.204, -0.048] |
| Frequency: Consolidation - Encoding | -0.029 | -0.923 | 0.793 | [-0.108, 0.051] |
| Frequency: Consolidation - Recollection | -0.090 | -2.950 | **0.050** | [-0.169, -0.012] |
| Frequency: Encoding - Recollection | -0.062 | -2.042 | 0.259 | [-0.14 - 0.016] |

Note: Linear mixed effects models with random intercept for each person, task stage, frequency and their interaction as fixed effect. Estimates indicate the unstandardized beta-coefficients. P-values are adjusted for multiple comparisons using the False Discovery rate.
