# Supplementary material for: Dynamic behavior of the locus coeruleus during arousal-related memory processing in a multi-modal 7T fMRI paradigm
Source: eLife. 2020 Jun 24;9:e52059. doi: 10.7554/eLife.52059 (PMC7343392; doi:10.7554/eLife.52059)
Supplement: Supplementary file 8. — Note: Brain regions activated during encoding, recollection or functional connectivity differences across the conditions (Family-wise error corrected for multiple comparisons using TFCE at p<0.05). Coordinates are provided in 1 mm MNI-space. [file elife-52059-supp8.docx]

**Supplementary File 8**: Task-related activation patterns during encoding and recollection and functional connectivity (FC) alterations across the task stages.

| **Region** | **Cluster volume**  **(mm)** | **Max Z-score or Min T-score (FC)** | **X** | **Y** | **Z** |
| --- | --- | --- | --- | --- | --- |
| 1. **Encoding: successful encoding > not successful encoding** | | | | | |
| Left Hippocampal tail | 44 | 3.83 | -33 | -35 | -5 |
| Right Basolateral Amygdala | 46 | 4.21 | 30 | -6 | -19 |
| Left Basolateral / centromedial Amygdala | 74 | 3.99 | -31 | -9 | -18 |
| Right Cornu Ammonis 1 | 175 | 4.22 | 35 | -13 | -17 |
| Right Entorhinal Cortex | 267 | 8.52 | 18 | -2 | -21 |
| Left Cornu Ammonis 3 | 353 | 5.97 | -22 | -17 | -14 |
| 1. **Recollection: successful recollection > not successful recollection** | | | | | |
| Right Subiculum | 44 | 4.32 | 24 | -27 | -9 |
| Left Cornu Ammonis 1 | 103 | 3.73 | -21 | -17 | -14 |
| 1. **Functional connectivity differences: consolidation < baseline (Spatially normalized)** | | | | | |
| No results after cluster-correction (for unadjusted results see Fig ) | | | | | |
| **Functional connectivity differences: consolidation < baseline (FIXed)** | | | | | |
| Left Parasubiculum | 3 | -5.16 | -18 | -14 | -23 |
| Left Superficial Amygdala | 5 | -4.12 | -16 | -10 | -20 |
| Left Basolateral Amygdala | 11 | -3.89 | -21 | -7 | -28 |
| Left Entorhinal Cortex | 29 | -3.89 | -17 | -3 | -26 |
| **Functional connectivity differences: consolidation < baseline (FIXed + Explicit Resp)** | | | | | |
| Left Superficial Amygdala | 10 | -4.52 | -19 | -1 | -17 |
| Left Parasubiculum | 31 | -5.32 | -18 | -14 | -23 |
| Left Basolateral Amygdala | 197 | -4.68 | -21 | -6 | -28 |
| **Functional connectivity differences: consolidation < baseline (FIXed + Explicit Phys)** | | | | | |
| Right Entorhinal cortex | 20 | -4.08 | 23 | 6 | -30 |
| Left Presubiculum | 30 | -5.03 | -18 | -14 | -23 |
| Left Basolateral Amygdala | 105 | -4.63 | -21 | -6 | -28 |

Note: Brain regions activated during encoding, recollection or functional connectivity differences across the conditions (Family-wise error corrected for multiple comparisons using TFCE at p<0.05). Coordinates are provided in 1mm MNI-space.
