# Supplementary material for: Dynamic behavior of the locus coeruleus during arousal-related memory processing in a multi-modal 7T fMRI paradigm
Source: eLife. 2020 Jun 24;9:e52059. doi: 10.7554/eLife.52059 (PMC7343392; doi:10.7554/eLife.52059)
Supplement: Transparent reporting form [file elife-52059-transrepform.docx]

***eLife’s* transparent reporting form**

We encourage authors to provide detailed information *within their submission* to facilitate the interpretation and replication of experiments. Authors can upload supporting documentation to indicate the use of appropriate reporting guidelines for health-related research (see [EQUATOR Network](http://www.equator-network.org/%20)), life science research (see the [BioSharing Information Resource](https://biosharing.org/)), or the [ARRIVE guidelines](http://www.plosbiology.org/article/info:doi/10.1371/journal.pbio.1000412) for reporting work involving animal research. Where applicable, authors should refer to any relevant reporting standards documents in this form.

If you have any questions, please consult our Journal Policies and/or contact us: [editorial@elifesciences.org](mailto:editorial@elifesciences.org).

**Sample-size estimation**

- You should state whether an appropriate sample size was computed when the study was being designed
- You should state the statistical method of sample size computation and any required assumptions
- If no explicit power analysis was used, you should describe how you decided what sample (replicate) size (number) to use

Please outline where this information can be found within the submission (e.g., sections or figure legends), or explain why this information doesn’t apply to your submission:

No explicit power analysis was conducted to determine sample size. Given the novelty of the approach and the focus on the brainstem, we were not able to determine a sample size a priori. However, the sample size is consistent with other recent brainstem studies (e.g. Bianciardi et al., 2016 MAGMA; Sclocco et al., 2016 Philos Trans A Math Phys Eng Sci). All data relating to the Figures 2-8 is provided in the data sources files. Inclusion criteria are described in the Methods. Final sample size for each element is described in the Methods (see also S-Table 1 and S-Table2) and is also indicated in each figure.

**Replicates**

- You should report how often each experiment was performed
- You should include a definition of biological versus technical replication
- The data obtained should be provided and sufficient information should be provided to indicate the number of independent biological and/or technical replicates
- If you encountered any outliers, you should describe how these were handled
- Criteria for exclusion/inclusion of data should be clearly stated
- High-throughput sequence data should be uploaded before submission, with a private link for reviewers provided (these are available from both GEO and ArrayExpress)

Please outline where this information can be found within the submission (e.g., sections or figure legends), or explain why this information doesn’t apply to your submission:

We replicated our findings using four different fMRI pipelines as described in the Methods and Results (technical replication). Pipelines differ in the extent of noise correction. For the fMRI data, outlier or noisy volumes were regressed out from the data as described in *Methods*. For the pulse rate data, adaptive thresholding was performed, excluding beats with physiologically improbable values. Inspection of data was performed using histograms, boxplots and q-q plots of residuals. If possible, statistical tests such as robust regression or bootstrapping were chosen.

**Statistical reporting**

- Statistical analysis methods should be described and justified
- Raw data should be presented in figures whenever informative to do so (typically when N per group is less than 10)
- For each experiment, you should identify the statistical tests used, exact values of N, definitions of center, methods of multiple test correction, and dispersion and precision measures (e.g., mean, median, SD, SEM, confidence intervals; and, for the major substantive results, a measure of effect size (e.g., Pearson's r, Cohen's d)
- Report exact p-values wherever possible alongside the summary statistics and 95% confidence intervals. These should be reported for all key questions and not only when the p-value is less than 0.05.

Please outline where this information can be found within the submission (e.g., sections or figure legends), or explain why this information doesn’t apply to your submission:

Statistical analyses are described in detail in the Methods section and concise descriptions are provided in the Results section, along with each analyses. Exact values of N (and number of observations), dispersion (median, IQR) and precision measures (median, confidence intervals) are provided in the Results section. Where possible, we also provide a measure of the effect size (correlation coefficients or t-stats). We report exact p-values for values >0.001 in the *Results* section for all primary analyses. Correction for multiple comparisons is described in the statistical section and for the fMRI analyses in the relevant method section.

(For large datasets, or papers with a very large number of statistical tests, you may upload a single table file with tests, Ns, etc., with reference to sections in the manuscript.)

**Group allocation**

- Indicate how samples were allocated into experimental groups (in the case of clinical studies, please specify allocation to treatment method); if randomization was used, please also state if restricted randomization was applied
- Indicate if masking was used during group allocation, data collection and/or data analysis

Please outline where this information can be found within the submission (e.g., sections or figure legends), or explain why this information doesn’t apply to your submission:

Not applicable

**Additional data files (“source data”)**

- We encourage you to upload relevant additional data files, such as numerical data that are represented as a graph in a figure, or as a summary table
- Where provided, these should be in the most useful format, and they can be uploaded as “Source data” files linked to a main figure or table
- Include model definition files including the full list of parameters used
- Include code used for data analysis (e.g., R, MatLab)
- Avoid stating that data files are “available upon request”

Please indicate the figures or tables for which source data files have been provided:

Numerical data for the figures 2-8 is provided in the Source data accompanying the specific figure (Rdata file types). We have not included data from the neuroimaging data (the fMRI data), given the limitations of our ethical approval (subjects did not consent for public sharing), but can be obtained from the authors.

Data analyses was performed in standard software packages that are publicly available (such as R, FSL, FreeSurfer, ANTs) or using functions in R or Matlab (equations are provided). This is listed in the Methods.
